# Serum lipids in adults with late age-related macular degeneration: a case-control study

**DOI:** 10.1186/s12944-018-0954-7

**Published:** 2019-01-08

**Authors:** Richard D. Semba, Ruin Moaddel, Mary Frances Cotch, Fridbert Jonasson, Gudny Eiriksdottir, Tamara B. Harris, Lenore J. Launer, Kai Sun, Ronald Klein, Debra A. Schaumberg, Pálmi Jónsson, Vilmundur Gudnason, Luigi Ferrucci

**Affiliations:** 1 Wilmer Eye Institute, Johns Hopkins University School of Medicine, Smith Building, M015, 400 N Broadway, Baltimore, MD 21287 USA; 2 Longitudinal Studies Section, National Institute on Aging, Baltimore, MD USA; 3 Division of Epidemiology and Clinical Research, National Eye Institute, Bethesda, MD USA; 4 Faculty of Medicine, University of Iceland, Reykjavik, Iceland; 5 Department of Ophthalmology, Landspitali University Hospital, Reykjavik, Iceland; 6 Icelandic Heart Association, Kopavogur, Iceland; 7 Laboratory of Epidemiology and Population Science, Intramural Research Program, National Institute on Aging, Bethesda, MD USA; 8 Department of Ophthalmology and Visual Sciences, University of Wisconsin School of Medicine and Public Health Madison, Madison, WI USA; 9 Moran Center for Translational Medicine, Department of Ophthalmology & Visual Sciences, University of Utah School of Medicine, Salt Lake City, UT USA

**Keywords:** Age-related macular degeneration, Aging, Geographic atrophy, Lipids, Mass spectrometry, Neovascular AMD

## Abstract

**Background:**

Lipids are implicated in the pathogenesis of age-related macular degeneration (AMD). The relationship between systemic lipids and AMD has not been well characterized. The objective was to investigate the relationship between serum lipids and AMD in older adults using a lipidomic approach.

**Methods:**

In a case-control study, 240 adults, aged ≥66 years, a third each having geographic atrophy, neovascular AMD, or no signs of AMD, were selected from a population-based sample of participants in the Age Gene/Environment Susceptibility-Reykjavik Study. The exposure was serum lipids and risk factors for AMD. The outcome was late AMD, assessed through fundus images taken through dilated pupils using a 45-degree digital camera and grading for neovascular AMD and geographic atrophy using the modified Wisconsin Age-Related Maculopathy Grading System.

**Results:**

Of 177 serum lipid species measured, there were no significant differences in serum lipids between controls and those with geographic atrophy or neovascular AMD, respectively. Adults with neovascular AMD had higher total serum lysophosphatidylcholine (LPC) (*P* = 0.004) and serum LPC 18:0 (*P* = 0.0002) compared to those with geographic atrophy.

**Conclusion:**

Late AMD was not characterized by alterations in systemic lipids compared with normal controls. These findings suggest that there may be differences in the LPC pathway between adults with neovascular AMD and geographic atrophy.

**Electronic supplementary material:**

The online version of this article (10.1186/s12944-018-0954-7) contains supplementary material, which is available to authorized users.

## Background

Age-related macular degeneration (AMD) is a leading cause of irreversible visual loss among older adults in developed countries [1]. Altered lipid metabolism is implicated in the pathogenesis of AMD. Lipid pathway genes, dietary lipid intake, and elevated high-density lipoprotein (HDL) are associated with increased risk of AMD [2]. AMD is characterized by early deposition of lipids in Bruch’s membrane and by drusen, focal deposits of extracellular debris composed of lipids [2]. Whether lipid abnormalities characteristic of AMD might be associated with alterations in systemic lipids is not well understood [2].

Lipids are the dominant biologic molecules in human serum, but relatively less is known about lipids because of their structural diversity and large number of species [3]. Recent advances in liquid chromatography-tandem mass spectrometry (LC-MS/MS) are facilitating new approaches towards revealing biomarkers and pathways associated with AMD [4]. We hypothesized that altered serum lipid concentrations are present in adults with late AMD. To address this hypothesis, we measured serum lipids in adults with and without late AMD using a lipidomics approach and LC-MS/MS.

## Methods

The Age, Gene/Environment Susceptibility Reykjavik Study (AGES) is a population-based study [5] that included AMD assessment, as described elsewhere [6]. Of 5764 adults examined in AGES (2002–2006), AMD status was determined in 5272 participants using fundus photography [6]. Retinal images were evaluated using the modified Wisconsin Age-related Maculopathy Grading System [6]. Of 5272 adults with AMD grading available, 3838 had no AMD, 1123 had early AMD, 133 had geographic atrophy (GA), and 178 had neovascular AMD. Simple random sampling was used to select 80 adults with no AMD, 80 with GA, and 80 with neovascular AMD.

Fasting venous blood was drawn, processed, and frozen at − 80 °C. Preanalytical studies show excellent long-term stability of serum lipids [7–9]. The Lipidyzer™ (Sciex, Framingham, MA) platform [10, 11] was used to measure lipids in the phosphatidylcholine (PC), phosphatidylethanolamine (PE), lysophosphatidylcholine (LPC), lysophosphatidylethanolamine (LPE), free fatty acid (FFA), sphingomyelin (SM), diacylglycerol (DAG), cholesteryl ester (CE), ceramide (CER), hexosylceramide (HCER), lactosylceramide (LCER), dihydroceramide (DCER), and triacylglycerol (TAG) groups of lipids on a 5500 QTrap® (Sciex) mass spectrometer. TAG and FFA were excluded from the analysis since the platform cannot differentiate TAG by specific acyl composition and FFA have poor reproducibility (Semba RD, et al., unpublished). Lipid species were included in the data analyses if above the limit of quantification in > 90% of the participants.

Continuous and categorical variables were compared across the three categories of AMD disease status using Kruskal-Wallis tests and chi-square tests, respectively. Serum lipid concentrations were log-transformed to achieve a normal distribution. A false discovery rate (FDR) approach was used to correct for multiple testing [11]. Q-values, the minimum FDR, was considered significant if *q* < 0.05. Univariable and multivariable logistic regression models were used to compare groups of lipids and individual lipid species between the three groups. Covariates with established associations with AMD such as age, smoking, and body mass index (BMI), and those associated with AMD in this study, including serum C-reactive protein (CRP) and chronic kidney disease, were included in the multivariable models. All analyses were performed using SAS (v. 9.1.3, SAS Institute, Inc., Cary, NC) with a type I error of 0.05 to determine statistical significance.

## Results

Characteristics of the study participants are shown in Table 1. Adults with GA and neovascular AMD were older (*P* < 0.0001) and had higher serum CRP concentrations (*P* = 0.01) and chronic kidney disease (*P* = 0.01) compared with controls. There were no significant differences between adults with GA or neovascular AMD and controls by sex, body mass index (BMI), smoking, total cholesterol, HDL cholesterol, LDL cholesterol, hypertension, coronary artery disease, or diabetes compared with controls.

**Table 1:** Characteristics of 240 participants, ≥66 years, in the AGES-Reykjavik Study.by AMD status

Characteristics^1^		AMD			*P*
		No AMD	Geographic Atrophy	Neovascular AMD	
		*n* = 80	*n* = 80	*n* = 80	
Age, y		75.0 (5.7)	82.3 (4.4)	81.7 (4.7)	< 0.0001
Sex, % women		55.0	60.0	56.3	0.80
Body mass index, kg/m^2^		27.1 (4.4)	26.5 (4.1)	26.3 (4.2)	0.46
Smoking, %	Never/Former	85.0	81.3	76.2	0.15
	Current	15.0	12.7	23.8	
Total cholesterol, mmol/L		5.6 (1.1)	5.5 (1.0)	5.7 (0.9)	0.50
HDL cholesterol, mmol/L		1.6 (0.5)	1.6 (0.5)	1.6 (0.5)	0.86
LDL cholesterol, mmol/L		3.5 (1.0)	3.4 (0.9)	3.5 (0.9)	0.62
Log C-reactive protein, mg/L		0.5 (1.0)	0.8 (1.1)	1.0 (1.0)	0.01
Hypertension, %		81.3	81.3	77.5	0.79
Coronary artery disease, %		36.4	49.3	42.9	0.31
Diabetes, %		15.0	18.8	7.5	0.11
Chronic kidney disease, %		40.9	45.2	64.3	0.01

^1^Conversion to SI units as follows: total cholesterol, HDL cholesterol, LDL cholesterol in mg/dL × 0.0259 = mmol/L; C-reactive protein in mg/L × 9.524 = μmol/L

A total of 177 serum lipids representing eleven groups of lipids (CER, HCER, LCER, DCER, CE, DAG, LPC, LPE, PC, PE, SM) were measured. Total mean serum concentrations of lipids in each group, adjusted by covariates, are shown in Table 2. Total serum LPC were higher in adults with neovascular AMD compared with GA (*P* = 0.004). There were no significant differences in total serum lipids in the other remaining lipid groups between controls and those with late AMD. Serum lipid species were compared between controls and those with GA or neovascular AMD, respectively. Mean serum lipid concentrations in controls versus those with GA or neovascular AMD, adjusted by covariates, are shown in Additional file 1: Table S1. Serum LPC 18:0 was higher in adults with neovascular AMD compared with GA (*P* = 0.0002).

**Table 2:** Total serum lipids in eight lipid classes in adults without AMD and adults with geographic atrophy or neovascular AMD^1^

Log total lipids^2^	No AMD	Geographic atrophy	Neovascular AMD	*P*	*P*	*P*
				Geographic atrophy vs no AMD	Neovascular AMD vs no AMD	Geographic atrophy vs neovascular AMD
CE	8.52 (0.04)	8.50 (0.04)	8.54 (0.04)	0.75	0.65	0.37
CER	2.16 (0.05)	2.10 (0.05)	2.11 (0.05)	0.43	0.39	0.75
DAG	3.68 (0.06)	3.67 (0.06)	3.63 (0.06)	0.90	0.52	0.78
DCER	0.41 (0.10)	0.23 (0.09)	0.25 (0.08)	0.20	0.22	0.69
HCER	1.87 (0.06)	1.77 (0.05)	1.83 (0.06)	0.29	0.59	0.38
LCER	2.08 (0.05)	1.94 (0.05)	1.97 (0.05)	0.07	0.29	0.57
LPC	5.67 (0.04)	5.64 (0.04)	5.78 (0.04)	0.61	0.05	0.004^3^
LPE	2.09 (0.07)	2.01 (0.07)	2.15 (0.06)	0.50	0.41	0.17
PC	7.94 (0.04)	8.00 (0.04)	7.98 (0.04)	0.27	0.65	0.73
PE	4.91 (0.08)	4.99 (0.07)	4.90 (0.07)	0.48	0.49	0.48
SM	6.89 (0.04)	6.92 (0.04)	6.94 (0.04)	0.69	0.46	0.64

^1^Adjusted by age, sex, smoking, BMI, CRP, and chronic kidney disease

^2^Log total serum lipids (μmol/L), showing mean (SE)

^3^Significant with q-value < 0.05

## Discussion

The present study showed no significant differences in serum concentrations of lipid species between adults with either GA or neovascular AMD compared with those without AMD. These findings suggest that the serum lipids measured in the present study are not associated with either form of late AMD. Whether abnormal deposits of retinal lipids in AMD is due to altered cellular lipid metabolism in the retinal pigment epithelium and neuroretina or from altered systemic lipids has not been completely clear [2, 12]. The present study does not support a strong role for altered systemic lipid species in the pathogenesis of AMD.

A recent study of 30 adults without AMD, 30 with early AMD, and 30 with late AMD used LC-MS/MS to identify circulating metabolites associated with AMD [4]. Several glycerophospholipid species were significantly decreased in AMD compared with controls. However, we were unable to corroborate these findings and found no significant differences in serum concentrations of DAG 18:0–20:4, DAG 18:1–18:1, DAG 18:1–18:2, PC 18:0–18:1, PC 18:2–20:4, and PC 18:0–20:4 in adults with GA or neovascular AMD, respectively, compared with controls. The previous study reported significant differences in these lipids between AMD and controls, however, the study did not adjust for multiple comparisons in the analysis involving nearly 700 plasma metabolites [4].

The present study shows serum LPC are higher in adults with neovascular AMD compared with GA. In the blood, LPC are primarily transported by albumin. LPC are biologically active lipids that serve as ligands for specific G protein-coupled signaling receptors and as precursors to lysophosphatidic acid (LPA), an intermediate in the biosynthetic pathway of cardiolipin [13, 14]. Cardiolipin is an important dimeric phospholipid found almost exclusively in mitochondria [14]. LPA also has specific receptors involved in growth, differentiation, and angiogenesis [15]. LPC pathway may distinguish neovascular AMD from GA in AMD pathogenesis.

The strengths of this study included the standardized assessment of AMD status, use of an advanced LC-MS/MS lipidomics platform, a conservative FDR approach in the statistical analysis, and comparison of normal controls with 80 adults with GA and 80 adults with neovascular AMD, instead of combining GA and neovascular AMD as a single category of late AMD as done in previous studies. The findings from AGES cannot necessarily be extrapolated to other people at risk of AMD. A limitation is that the study did not include adults with early AMD and thus does not exclude the possibility that systemic lipid alterations might occur in the earlier stages of AMD.

## Conclusions

The present study suggests that serum lipids are not associated with late AMD. Future studies are needed to determine whether other lipid classes not covered by the present study might be associated with the prevalence or progression of AMD.

## Additional file

Additional file 1: **Table S1.** Serum lipids species in adults without AMD and adults with geographic atrophy or neovascular AMD^1^. (DOCX 31 kb)

## References

[1] Wong WL, Su X, Li X (2014). Global prevalence of age-related macular degeneration and disease burden projection for 2020 and 2040: a systematic review and meta-analysis. Lancet Glob Health.

[2] van Leeuwen EM, Emri E, Merle BMJ, et al. A new perspective on lipid research in age-related macular degeneration. Prog Retin Eye Res. 2018 May 4. pii: S1350–9462(17)30127–1. 10.1016/j.preteyeres.2018.04.006 29729972

[3] Quehenberger O, Dennis EA (2011). The human plasma lipidome. N Engl J Med.

[4] Laíns I, Kelly RS, Miller JB (2018). Human plasma metabolomics study across all stages of age-related macular degeneration identifies potential lipid biomarkers. Ophthalmology.

[5] Harris TB, Launer LJ, Eiriksdottir G (2007). Age, gene/environment susceptibility-Reykjavik study: multidisciplinary applied phenomics. Am J Epidemiol.

[6] Jonasson F, Arnarsson A, Eiríksdottir G (2011). Prevalence of age-related macular degeneration in older persons: age, Gene/Environment Susceptibility Reykjavik Study. Ophthalmology.

[7] Heiskanen LA, Suoniemi M, Ta HX, Tarasov K, Ekroos K (2013). Long-term performance and stability of molecular shotgun lipidomic analysis of human plasma samples. Anal Chem.

[8] Matthan NR, Ip B, Resteghini N, Ausman LM, Lichtenstein AH (2010). Long-term fatty acid stability in human serum cholesteryl ester, triglyceride, and phospholipid fractions. J Lipid Res.

[9] Surma MA, Herzog R, Vasilj A (2015). An automated shotgun lipidomics platform for high throughput, comprehensive, and quantitative analysis of blood plasma intact lipids. Eur J Lipid Sci Technol.

[10] Baker PR, Armando AM, Campbell JL, Quehenberger O, Dennis EA (2014). Three-dimensional enhanced lipidomics analysis combining UPLC, differential ion mobility spectrometry, and mass spectrometric separation strategies. J Lipid Res.

[11] Storey JD, Taylor JE, Siegmund D (2004). Strong control, conservative point estimation, and simultaneous conservative consistency of false discovery rates: a unified approach. J Roy Stat Soc Ser B.

[12] Curcio CA (2018). Soft drusen in age-related macular degeneration: biology and targeting via the oil spill strategies. Invest Ophthalmol Vis Sci.

[13] Grzelczyk A, Gendaszewska-Darmach E (2013). Novel bioactive glycerol-based lysophospholipids: new data -- new insight into their function. Biochimie.

[14] Schlame M, Greenberg ML (1862). Biosynthesis, remodeling and turnover of mitochondrial cardiolipin. Biochim Biophys Acta.

[15] Benesch MG, Tang X, Venkatraman G, Bekele RT, Brindley DN (2016). Recent advances in targeting the autotaxin-lysophosphatidate-lipid phosphate phosphatase axis in vivo. J Biomed Res.

