# Supplementary material for: Serum lipids in adults with late age-related macular degeneration: a case-control study
Source: Lipids Health Dis. 2019 Jan 8;18:7. doi: 10.1186/s12944-018-0954-7 (PMC6323843; doi:10.1186/s12944-018-0954-7)
Supplement: Supplementary file 1 — Table S1. Serum lipids species in adults without AMD and adults with geographic atrophy or neovascular AMD1. (DOCX 31 kb) [file 12944_2018_954_MOESM1_ESM.docx]

**Table S1 Serum lipids species in adults without AMD and adults with geographic atrophy or neovascular AMD^1^**

| **Log lipid species^2^** | **No AMD** | **Geographic atrophy** | **Neovascular AMD** | ***P***  **Geographic atrophy vs no AMD** | ***P***  **Neovascular AMD vs**  **no AMD** | ***P***  **Geographic atrophy vs neovascular AMD** |
| --- | --- | --- | --- | --- | --- | --- |
| CE 14:0 | 3.90 (0.05) | 3.94 (0.05) | 3.90 (0.05) | 0.60 | 0.96 | 0.95 |
| CE 14:1 | 0.84 (0.07) | 0.89 (0.07) | 0.79 (0.06) | 0.66 | 0.55 | 0.44 |
| CE 15:0 | 2.81 (0.05) | 2.87 (0.05) | 2.77 (0.05) | 0.47 | 0.33 | 0.58 |
| CE 16:0 | 6.34 (0.04) | 6.36 (0.04) | 6.38 (0.04) | 0.82 | 0.68 | 0.42 |
| CE 16:1 | 5.26 (0.05) | 5.23 (0.05) | 5.26 (0.05) | 0.70 | 0.62 | 0.85 |
| CE 17:0 | 3.02 (0.04) | 3.02 (0.05) | 2.99 (0.04) | 0.96 | 0.66 | 0.85 |
| CE 18:0 | 4.39 (0.04) | 4.38 (0.04) | 4.40 (0.04) | 0.88 | 0.74 | 0.75 |
| CE 18:1 | 6.88 (0.04) | 6.87 (0.04) | 6.91 (0.03) | 0.89 | 0.66 | 0.53 |
| CE 18:2 | 7.71 (0.04) | 7.72 (0.04) | 7.76 (0.04) | 0.91 | 0.47 | 0.38 |
| CE 18:3 | 4.16 (0.05) | 4.23 (0.05) | 4.23 (0.05) | 0.41 | 0.89 | 0.35 |
| CE 18:4 | 0.99 (0.08) | 1.21 (0.08) | 1.07 (0.08) | 0.11 | 0.40 | 0.35 |
| CE 20:0 | 1.52 (0.14) | 1.88 (0.15) | 1.81 (0.15) | 0.13 | 0.88 | 0.11 |
| CE 20:1 | 1.26 (0.18) | 1.75 (0.19) | 1.53 (0.19) | 0.10 | 0.88 | 0.17 |
| CE 20:2 | 1.16 (0.08) | 1.29 (0.08) | 1.22 (0.08) | 0.33 | 0.96 | 0.52 |
| CE 20:3 | 3.60 (0.05) | 3.64 (0.05) | 3.61 (0.05) | 0.62 | 0.97 | 0.73 |
| CE 20:4 | 5.55 (0.04) | 5.65 (0.04) | 5.59 (0.04) | 0.17 | 0.54 | 0.34 |
| CE 20:5 | 5.02 (0.09) | 5.17 (0.09) | 5.07 (0.09) | 0.30 | 0.60 | 0.39 |
| CE 22:0 | 0.79 (0.11) | 1.12 (0.12) | 0.89 (0.11) | 0.07 | 0.38 | 0.22 |
| CE 22:1 | 0.17 (0.11) | 0.46 (0.12) | 0.32 (0.11) | 0.11 | 0.81 | 0.24 |
| CE 22:2 | 0.07 (0.20) | 0.64 (0.21) | 0.46 (0.20) | 0.08 | 0.94 | 0.12 |
| CE 22:4 | 0.57 (0.28) | 1.37 (0.29) | 0.94 (0.28) | 0.07 | 0.75 | 0.19 |
| CE 22:5 | 2.00 (0.07) | 2.17 (0.07) | 2.07 (0.07) | 0.16 | 0.61 | 0.28 |
| CE 22:6 | 4.36 (0.06) | 4.46 (0.06) | 4.39 (0.06) | 0.28 | 0.63 | 0.36 |
| CE 24:1 | -0.10 (0.18) | 0.44 (0.19) | 0.21 (0.18) | 0.06 | 0.85 | 0.11 |
| CER 16:0 | -0.45 (0.07) | -0.27 (0.07) | -0.37 (0.08) | 0.12 | 0.36 | 0.17 |
| CER 18:0 | -1.66 (0.05) | -1.61 (0.05) | -1.67 (0.05) | 0.48 | 0.47 | 0.99 |
| CER 20:0 | -1.80 (0.04) | -1.76 (0.04) | -1.81 (0.04) | 0.51 | 0.46 | 0.99 |
| CER 22:0 | 0.11 (0.04) | 0.10 (0.04) | 0.12 (0.04) | 0.84 | 0.86 | 0.88 |
| CER 22:1 | -2.21 (0.05) | -2.19 (0.05) | -2.20 (0.04) | 0.83 | 0.94 | 0.95 |
| CER 24:0 | 1.27 (0.04) | 1.28 (0.05) | 1.27 (0.04) | 0.85 | 0.80 | 0.99 |
| CER 24:1 | 0.73 (0.04) | 0.71 (0.04) | 0.72 (0.03) | 0.80 | 0.79 | 0.82 |
| DAG 14:0-18:1 | -0.20 (0.07) | -0.22 (0.08) | -0.26 (0.07) | 0.86 | 0.78 | 0.66 |
| DAG 14:1-18:1 | -0.35 (0.09) | -0.57 (0.10) | -0.54 (0.09) | 0.18 | 0.99 | 0.45 |
| DAG 16:0-16:0 | -0.26 (0.07) | -0.18 (0.08) | -0.28 (0.08) | 0.53 | 0.30 | 0.79 |
| DAG 16:0-16:1 | -0.37 (0.06) | -0.38 (0.07) | -0.42 (0.06) | 0.94 | 0.68 | 0.69 |
| DAG 16:0-18:0 | -0.19 (0.06) | -0.07 (0.07) | -0.18 (0.07) | 0.26 | 0.24 | 0.49 |
| DAG 16:0-18:1 | 1.52 (0.06) | 1.48 (0.06) | 1.51 (0.06) | 0.72 | 0.90 | 0.72 |
| DAG 16:0-18:2 | 0.59 (0.07) | 0.58 (0.07) | 0.60 (0.08) | 0.87 | 0.90 | 0.55 |
| DAG 16:0-22:6 | -1.44 (0.08) | -1.16 (0.09) | -1.44 (0.08) | 0.04 | 0.06 | 0.54 |
| DAG 16:1-18:1 | 0.80 (0.06) | 0.67 (0.06) | 0.67 (0.06) | 0.20 | 0.81 | 0.22 |
| DAG 16:1-18:2 | -0.25 (0.07) | -0.34 (0.08) | -0.37 (0.076) | 0.46 | 0.73 | 0.34 |
| DAG 16:1-22:6 | -1.75 (0.09) | -1.56 (0.09) | -1.83 (0.088) | 0.20 | 0.13 | 0.63 |
| DAG 18:0-18:1 | 0.29 (0.06) | 0.22 (0.06) | 0.23 (0.061) | 0.49 | 0.84 | 0.76 |
| DAG 18:0-18:2 | -0.50 (0.07) | -0.54 (0.08) | -0.55 (0.08) | 0.74 | 0.80 | 0.91 |
| DAG 18:1-18:1 | 1.92 (0.05) | 1.80 (0.06) | 1.85 (0.06) | 0.23 | 0.99 | 0.62 |
| DAG 18:1-18:2 | 1.64 (0.06) | 1.50 (0.06) | 1.57 (0.07) | 0.15 | 0.81 | 0.72 |
| DAG 18:1-20:4 | -0.95 (0.06) | -0.83 (0.06) | -0.96 (0.06) | 0.24 | 0.17 | 0.82 |
| DAG 18:1-20:5 | -1.05 (0.08) | -0.78 (0.08) | -0.97 (0.08) | 0.05 | 0.24 | 0.24 |
| DAG 18:1-22:5 | -1.51 (0.06) | -1.38 (0.06) | -1.46 (0.06) | 0.21 | 0.48 | 0.22 |
| DAG 18:1-22:6 | -0.39 (0.08) | -0.19 (0.09) | -0.45 (0.08) | 0.15 | 0.03 | 0.94 |
| DAG 18:2-20:4 | -1.67 (0.06) | -1.52 (0.07) | -1.66 (0.07) | 0.17 | 0.29 | 0.70 |
| DAG 18:2-22:6 | -1.26 (0.09) | -0.99 (0.09) | -1.19 (0.08) | 0.06 | 0.29 | 0.30 |
| DAG 20:0-20:0 | 0.54 (0.14) | 0.86 (0.15) | 0.82 (0.14) | 0.16 | 0.81 | 0.07 |
| DCER 24:0 | -0.98 (0.05) | -0.94 (0.05) | -0.98 (0.04) | 0.58 | 0.60 | 0.98 |
| DCER 24:1 | -0.85 (0.03) | -0.82 (0.04) | -0.88 (0.03) | 0.64 | 0.30 | 0.69 |
| HCER 16:0 | 1.15 (0.22) | 0.98 (0.24) | 0.92 (0.22) | 0.63 | 0.66 | 0.79 |
| HCER 18:0 | -1.10 (0.14) | -1.23 (0.15) | -1.25 (0.14) | 0.58 | 0.71 | 0.88 |
| HCER 20:0 | -0.68 (0.26) | -0.95 (0.28) | -0.93 (0.25) | 0.52 | 0.84 | 0.83 |
| HCER 22:0 | 0.64 (0.15) | 0.47 (0.16) | 0.49 (0.15) | 0.51 | 0.86 | 0.79 |
| HCER 22:1 | -0.77 (0.04) | -0.78 (0.04) | -0.82 (0.04) | 0.85 | 0.57 | 0.39 |
| HCER 24:0 | 0.01 (0.09) | 0.31 (0.09) | 0.12 (0.08) | 0.04 | 0.30 | 0.78 |
| HCER 24:1 | -0.02 (0.07) | 0.07 (0.08) | 0.12 (0.07) | 0.43 | 0.54 | 0.18 |
| LCER 14:0 | -0.69 (0.11) | -0.75 (0.12) | -0.85 (0.11) | 0.75 | 0.50 | 0.55 |
| LCER 16:0 | 1.02 (0.08) | 1.16 (0.09) | 1.09 (0.08) | 0.31 | 0.68 | 0.81 |
| LCER 18:1 | -0.48 (0.04) | -0.35 (0.04) | -0.39 (0.03) | 0.04 | 0.67 | 0.10 |
| LCER 20:1 | -0.97 (0.09) | -0.95 (0.09) | -0.96 (0.09) | 0.85 | 0.93 | 0.32 |
| LCER 22:0 | -1.38 (0.09) | -0.98 (0.10) | -1.17 (0.09) | 0.01 | 0.49 | 0.09 |
| LCER 24:0 | -0.87 (0.11) | -0.89 (0.11) | -0.91 (0.10) | 0.89 | 0.84 | 0.66 |
| LCER 24:1 | -0.61 (0.05) | -0.50 (0.06) | -0.53 (0.05) | 0.25 | 0.96 | 0.15 |
| LPC 14:0 | -0.46 (0.05) | -0.40 (0.06) | -0.41 (0.05) | 0.56 | 0.68 | 0.44 |
| LPC 15:0 | 0.15 (0.04) | 0.22 (0.04) | 0.17 (0.04) | 0.31 | 0.71 | 0.43 |
| LPC 16:0 | 5.04 (0.04) | 5.09 (0.04) | 5.19 (0.03) | 0.44 | 0.06 | 0.001 |
| LPC 16:1 | 1.41 (0.04) | 1.40 (0.04) | 1.50 (0.04) | 0.81 | 0.13 | 0.11 |
| LPC 17:0 | 1.22 (0.04) | 1.26 (0.04) | 1.29 (0.04) | 0.66 | 0.51 | 0.08 |
| LPC 18:0 | 3.86 (0.04) | 3.89 (0.04) | 4.03 (0.04) | 0.64 | 0.01 | 0.0002^3^ |
| LPC 18:1 | 3.46 (0.04) | 3.44 (0.04) | 3.56 (0.04) | 0.82 | 0.06 | 0.06 |
| LPC 18:2 | 3.27 (0.05) | 3.24 (0.05) | 3.34 (0.05) | 0.74 | 0.13 | 0.52 |
| LPC 20:3 | 0.67 (0.06) | 0.62 (0.06) | 0.71 (0.05) | 0.62 | 0.36 | 0.81 |
| LPC 20:4 | 1.24 (0.06) | 1.34 (0.07) | 1.34 (0.07) | 0.31 | 0.85 | 0.20 |
| LPC 20:5 | -0.21 (0.07) | -0.09 (0.06) | -0.14 (0.08) | 0.33 | 0.85 | 0.25 |
| LPE 16:0 | 0.26 (0.06) | 0.35 (0.06) | 0.36 (0.05) | 0.37 | 0.78 | 0.19 |
| LPE 18:0 | 0.68 (0.05) | 0.77 (0.06) | 0.81 (0.05) | 0.34 | 0.54 | 0.06 |
| LPE 18:1 | 0.30 (0.07) | 0.34 (0.08) | 0.44 (0.07) | 0.74 | 0.23 | 0.24 |
| LPE 18:2 | 0.68 (0.09) | 0.69 (0.09) | 0.82 (0.08) | 0.91 | 0.21 | 0.38 |
| LPE 20:4 | -1.00 (0.10) | -0.74 (0.11) | -0.79 (0.10) | 0.11 | 0.97 | 0.16 |
| PC 14:0-18:1 | 2.23 (0.05) | 2.20 (0.05) | 2.17 (0.05) | 0.71 | 0.72 | 0.42 |
| PC 14:0-18:2 | 2.13 (0.05) | 2.10 (0.06) | 2.06 (0.06) | 0.74 | 0.78 | 0.32 |
| PC 14:0-20:3 | 0.61 (0.07) | 0.62 (0.07) | 0.55 (0.07) | 0.91 | 0.77 | 0.66 |
| PC 14:0-20:4 | 1.48 (0.07) | 1.55 (0.08) | 1.42 (0.07) | 0.59 | 0.36 | 0.64 |
| PC 15:0-18:1 | 0.66 (0.04) | 0.63 (0.05) | 0.56 (0.04) | 0.67 | 0.37 | 0.11 |
| PC 15:0-18:2 | 0.74 (0.04) | 0.71 (0.04) | 0.63 (0.04) | 0.71 | 0.26 | 0.09 |
| PC 16:0-12:0 | -0.16 (0.06) | -0.26 (0.06) | -0.23 (0.06) | 0.31 | 0.85 | 0.61 |
| PC 16:0-14:0 | 1.57 (0.06) | 1.53 (0.06) | 1.51 (0.06) | 0.69 | 0.93 | 0.39 |
| PC 16:0-16:0 | 4.49 (0.07) | 4.31 (0.07) | 4.51 (0.07) | 0.10 | 0.11 | 0.78 |
| PC 16:0-16:1 | 3.16 (0.05) | 3.04 (0.05) | 3.06 (0.06) | 0.19 | 0.97 | 0.18 |
| PC 16:0-18:0 | 4.45 (0.07) | 4.27 (0.07) | 4.49 (0.07) | 0.11 | 0.08 | 0.51 |
| PC 16:0-18:1 | 6.07 (0.04) | 6.00 (0.04) | 6.04 (0.04) | 0.24 | 0.75 | 0.64 |
| PC 16:0-18:2 | 6.54 (0.04) | 6.44 (0.04) | 6.51 (0.04) | 0.16 | 0.41 | 0.53 |
| PC 16:0-18:3 | 2.57 (0.05) | 2.49 (0.06) | 2.51 (0.06) | 0.39 | 0.85 | 0.40 |
| PC 16:0-20:1 | 1.37 (0.06) | 1.31 (0.06) | 1.34 (0.06) | 0.53 | 0.69 | 0.94 |
| PC 16:0-20:2 | 2.24 (0.04) | 2.10 (0.04) | 2.16 (0.04) | 0.04 | 0.55 | 0.17 |
| PC 16:0-20:3 | 4.55 (0.05) | 4.46 (0.05) | 4.48 (0.05) | 0.30 | 0.84 | 0.38 |
| PC 16:0-20:4 | 5.32 (0.04) | 5.35 (0.04) | 5.28 (0.04) | 0.70 | 0.39 | 0.62 |
| PC 16:0-20:5 | 4.43 (0.08) | 4.55 (0.08) | 4.42 (0.08) | 0.37 | 0.27 | 0.78 |
| PC 16:0-22:4 | 1.73 (0.05) | 1.73 (0.05) | 1.73 (0.06) | 0.94 | 0.97 | 0.96 |
| PC 16:0-22:5 | 3.88 (0.04) | 3.89 (0.05) | 3.88 (0.04) | 0.87 | 0.61 | 0.75 |
| PC 16:0-22:6 | 5.55 (0.05) | 5.54 (0.05) | 5.53 (0.05) | 0.88 | 0.79 | 0.85 |
| PC 17:0-18:1 | 1.48 (0.04) | 1.38 (0.05) | 1.38 (0.04) | 0.18 | 0.72 | 0.24 |
| PC 17:0-18:2 | 1.98 (0.04) | 1.91 (0.04) | 1.90 (0.04) | 0.23 | 0.61 | 0.25 |
| PC 17:0-20:3 | 0.59 (0.05) | 0.49 (0.05) | 0.47 (0.05) | 0.23 | 0.86 | 0.12 |
| PC 17:0-20:4 | 1.17 (0.05) | 1.16 (0.05) | 1.06 (0.05) | 0.85 | 0.18 | 0.19 |
| PC 17:0-22:6 | 0.33 (0.06) | 0.31 (0.07) | 0.19 (0.06) | 0.89 | 0.16 | 0.23 |
| PC 18:0-14:0 | -0.19 (0.06) | -0.17 (0.07) | -0.21 (0.06) | 0.87 | 0.90 | 0.96 |
| PC 18:0-16:1 | 0.94 (0.06) | 0.81 (0.06) | 0.87 (0.06) | 0.18 | 0.79 | 0.39 |
| PC 18:0-18:0 | 1.38 (0.05) | 1.21 (0.05) | 1.37 (0.06) | 0.06 | 0.14 | 0.92 |
| PC 18:0-18:1 | 4.13 (0.04) | 3.99 (0.05) | 4.09 (0.04) | 0.08 | 0.32 | 0.69 |
| PC 18:0-18:2 | 5.53 (0.04) | 5.41 (0.045) | 5.51 (0.04) | 0.07 | 0.20 | 0.76 |
| PC 18:0-18:3 | 1.24 (0.06) | 1.15 (0.06) | 1.23 (0.06) | 0.38 | 0.49 | 0.90 |
| PC 18:0-20:0 | 0.17 (0.05) | 0.14 (0.05) | 0.13 (0.05) | 0.69 | 0.71 | 0.80 |
| PC 18:0-20:1 | -0.06 (0.07) | -0.13 (0.07) | -0.06 (0.07) | 0.56 | 0.42 | 0.79 |
| PC 18:0-20:2 | 1.04 (0.05) | 0.86 (0.05) | 0.98 (0.05) | 0.03 | 0.24 | 0.37 |
| PC 18:0-20:3 | 3.47 (0.05) | 3.36 (0.05) | 3.41 (0.05) | 0.18 | 0.54 | 0.50 |
| PC 18:0-20:4 | 4.39 (0.04) | 4.40 (0.04) | 4.37 (0.04) | 0.88 | 0.62 | 0.88 |
| PC 18:0-20:5 | 3.54 (0.08) | 3.61 (0.09) | 3.53 (0.08) | 0.58 | 0.53 | 0.68 |
| PC 18:0-22:5 | 2.43 (0.05) | 2.42 (0.05) | 2.45 (0.04) | 0.96 | 0.86 | 0.47 |
| PC 18:0-22:6 | 4.12 (0.05) | 4.08 (0.06) | 4.10 (0.05) | 0.68 | 0.93 | 0.77 |
| PC 18:1-16:1 | 2.75 (0.04) | 2.63 (0.04) | 2.68 (0.04) | 0.09 | 0.85 | 0.34 |
| PC 18:1-18:1 | 3.22 (0.04) | 3.07 (0.05) | 3.18 (0.04) | 0.04 | 0.27 | 0.63 |
| PC 18:1-18:2 | 4.40 (0.04) | 4.26 (0.04) | 4.37 (0.04) | 0.04 | 0.21 | 0.55 |
| PC 18:1-18:3 | 0.66 (0.06) | 0.57 (0.06) | 0.61 (0.06) | 0.34 | 0.75 | 0.55 |
| PC 18:1-20:2 | 0.10 (0.05) | -0.04 (0.05) | 0.05 (0.05) | 0.08 | 0.25 | 0.38 |
| PC 18:1-20:3 | 2.20 (0.05) | 2.04 (0.06) | 2.11 (0.06) | 0.08 | 0.54 | 0.33 |
| PC 18:1-20:4 | 3.05 (0.04) | 3.03 (0.05) | 3.02 (0.05) | 0.75 | 0.82 | 0.64 |
| PC 18:1-20:5 | 2.20 (0.08) | 2.26 (0.08) | 2.15 (0.07) | 0.65 | 0.30 | 0.96 |
| PC 18:1-22:6 | 2.57 (0.05) | 2.50 (0.05) | 2.53 (0.05) | 0.37 | 0.89 | 0.94 |
| PC 18:2-16:1 | 2.78 (0.04) | 2.65 (0.04) | 2.70 (0.04) | 0.06 | 0.77 | 0.18 |
| PC 18:2-18:2 | 2.55 (0.05) | 2.42 (0.06) | 2.59 (0.06) | 0.19 | 0.10 | 0.70 |
| PC 18:2-20:1 | -0.01 (0.05) | -0.05 (0.06) | -0.03 (0.06) | 0.57 | 0.73 | 0.86 |
| PC 18:2-20:3 | 0.55 (0.05) | 0.47 (0.06) | 0.53 (0.06) | 0.41 | 0.48 | 0.84 |
| PC 18:2-20:4 | 1.55 (0.04) | 1.55 (0.05) | 1.56 (0.05) | 0.96 | 0.98 | 0.76 |
| PC 20:0-18:1 | 0.27 (0.05) | 0.21 (0.06) | 0.27 (0.05) | 0.53 | 0.55 | 0.79 |
| PC 20:0-18:2 | 0.93 (0.04) | 0.84 (0.05) | 0.90 (0.05) | 0.28 | 0.63 | 0.73 |
| PC 20:0-20:4 | 0.31 (0.05) | 0.31 (0.05) | 0.24 (0.05) | 0.98 | 0.34 | 0.67 |
| PE 16:0-18:1 | 0.13 (0.06) | 0.12 (0.07) | 0.03 (0.06) | 0.88 | 0.35 | 0.28 |
| PE 16:0-18:2 | 1.00 (0.07) | 0.84 (0.07) | 0.87 (0.07) | 0.17 | 0.89 | 0.15 |
| PE 16:0-20:3 | -0.97 (0.07) | -1.01 (0.08) | -1.09 (0.08) | 0.75 | 0.46 | 0.40 |
| PE 16:0-20:4 | 1.05 (0.07) | 0.97 (0.07) | 0.91 (0.07) | 0.47 | 0.57 | 0.21 |
| PE 16:0-22:6 | 2.58 (0.07) | 2.57 (0.07) | 2.43 (0.07) | 0.94 | 0.10 | 0.32 |
| PE 18:0-18:1 | 1.27 (0.05) | 1.23 (0.05) | 1.24 (0.05) | 0.62 | 0.77 | 0.84 |
| PE 18:0-18:2 | 2.35 (0.05) | 2.24 (0.06) | 2.30 (0.06) | 0.25 | 0.80 | 0.47 |
| PE 18:0-20:3 | 0.56 (0.06) | 0.47 (0.06) | 0.46 (0.06) | 0.35 | 0.82 | 0.33 |
| PE 18:0-20:4 | 2.09 (0.05) | 2.09 (0.06) | 2.00 (0.06) | 0.99 | 0.31 | 0.35 |
| PE 18:0-22:6 | 2.38 (0.06) | 2.39 (0.06) | 2.28 (0.06) | 0.91 | 0.14 | 0.64 |
| PE 18:1-18:1 | -0.79 (0.06) | -0.91 (0.06) | -0.88 (0.06) | 0.24 | 0.74 | 0.25 |
| PE 18:1-18:2 | 0.52 (0.07) | 0.34 (0.07) | 0.42 (0.07) | 0.11 | 0.56 | 0.20 |
| PE 18:1-20:4 | 0.40 (0.06) | 0.28 (0.06) | 0.24 (0.06) | 0.27 | 0.60 | 0.09 |
| PEO 16:0-20:4 | -0.29 (0.06) | -0.26 (0.07) | -0.32 (0.06) | 0.77 | 0.53 | 0.74 |
| PEO 16:0-22:6 | 0.82 (0.05) | 0.86 (0.05) | 0.78 (0.05) | 0.59 | 0.23 | 0.95 |
| PEO 18:0-18:1 | -1.22 (0.04) | -1.30 (0.05) | -1.26 (0.04) | 0.31 | 0.62 | 0.75 |
| PEO 18:0-18:2 | -0.55 (0.05) | -0.66 (0.06) | -0.58 (0.05) | 0.24 | 0.63 | 0.86 |
| PEO 18:0-20:4 | 0.20 (0.05) | 0.15 (0.06) | 0.09 (0.05) | 0.58 | 0.40 | 0.25 |
| PEO 18:0-22:6 | 0.73 (0.05) | 0.70 (0.05) | 0.64 (0.05) | 0.79 | 0.25 | 0.61 |
| PEP 18:0-18:1 | 0.19 (0.09) | 0.15 (0.10) | 0.20 (0.08) | 0.80 | 0.96 | 0.84 |
| PEP 18:0-18:2 | 1.49 (0.13) | 1.23 (0.13) | 1.26 (0.14) | 0.20 | 0.97 | 0.15 |
| PEP 18:0-20:4 | 2.04 (0.13) | 1.89 (0.14) | 1.87 (0.13) | 0.47 | 0.56 | 0.35 |
| SM 14:0 | 3.69 (0.04) | 3.70 (0.04) | 3.70 (0.04) | 0.89 | 0.79 | 0.99 |
| SM 16:0 | 5.82 (0.03) | 5.80 (0.04) | 5.84 (0.03) | 0.69 | 0.51 | 0.71 |
| SM 18:0 | 4.06 (0.04) | 4.04 (0.04) | 4.07 (0.04) | 0.79 | 0.78 | 0.81 |
| SM 18:1 | 3.19 (0.04) | 3.15 (0.04) | 3.16 (0.04) | 0.52 | 0.85 | 0.67 |
| SM 20:0 | 5.08 (0.06) | 5.09 (0.07) | 5.13 (0.06) | 0.89 | 0.61 | 0.55 |
| SM 20:1 | 3.29 (0.04) | 3.19 (0.04) | 3.25 (0.05) | 0.20 | 0.76 | 0.48 |
| SM 22:0 | 4.79 (0.05) | 4.77 (0.05) | 4.83 (0.04) | 0.84 | 0.39 | 0.47 |
| SM 22:1 | 4.30 (0.04) | 4.22 (0.04) | 4.29 (0.04) | 0.20 | 0.41 | 0.82 |
| SM 24:0 | 3.69 (0.03) | 3.67 (0.03) | 3.71 (0.03) | 0.62 | 0.47 | 0.76 |
| SM 24:1 | 4.53 (0.04) | 4.39 (0.04) | 4.50 (0.04) | 0.05 | 0.30 | 0.62 |
| SM 26:0 | 1.24 (0.08) | 1.42 (0.08) | 1.35 (0.08) | 0.17 | 0.96 | 0.22 |
| SM 26:1 | 1.38 (0.06) | 1.54 (0.06) | 1.48 (0.06) | 0.13 | 0.96 | 0.14 |

^1^Adjusted by age, sex, smoking, BMI, CRP, and chronic kidney disease.

^2^Log total serum lipids (µmol/L), showing mean (SE).

^3^Significant with q-value < 0.05
